# The effect of cavernous nerve traction on erectile function in rats

**DOI:** 10.1371/journal.pone.0186077

**Published:** 2017-10-05

**Authors:** Hao Li, Liping Chen, Tao Wang, Shaogang Wang, Jihong Liu

**Affiliations:** 1 Department of Urology, Tongji Hospital, Tongji Medical College, Huazhong University of Science and Technology, Wuhan, China; 2 Institute of Urology, Tongji Hospital, Tongji Medical College, Huazhong University of Science and Technology, Wuhan, China; 3 Department of Gastroenterology, Tongji Hospital, Tongji Medical College, Huazhong University of Science and Technology, Wuhan, China; University of Miami School of Medicine, UNITED STATES

## Abstract

We performed this study to evaluate the effect of cavernous nerve (CN) traction on erectile function in rats. Thirty-two 8- week-old Sprague–Dawley rats were divided into four groups: control, 1-minute CN traction, 2-minute CN traction, and 2-minute CN crush. CN traction was performed using a glass hook with a tensile force of 0.2 Newton. One month later, the mean arterial pressure (MAP) and intracavernosal pressure (ICP) in response to CN stimulation were measured to assess erectile function. The penis and major pelvic ganglion (MPG) were harvested to explore the expression of neuronal nitric oxide synthase (nNOS) and neurofilament, fibrosis and apoptosis. The ICP/MAP ratio was reduced in the 2-minute CN traction group compared with the control group (P < 0.05). The ICP/MAP ratio in the CN crush group was lower than in the other three groups (P < 0.05, for each). Expression of nNOS in both MPG and dorsal penile nerve was lower in the CN traction group than in the control group, but was higher than in the CN crush group (P < 0.05). Nerve fiber number in the dorsal penile nerve was reduced by 2-minute CN traction (P < 0.05). The ratios of collagen to smooth muscle content and the apoptosis were both increased the in 2-minute CN traction group compared with the control group (P < 0.05). The findings indicate that CN traction is an effective CN injury model and the injury it caused is relatively mild compared with the CN crush model.

## Introduction

Iatrogenic cavernous nerve (CN) injuries resulting from some types of pelvic surgeries, such as abdominoperineal resection, radical prostatectomy (RP), and radical cystoprostatectomy, often lead to severe erectile dysfunction (ED) [1]. It is estimated that 26% to 100% patients suffer from ED after RP [2]. The main reason for this complication is the injury to the penile nerve supply [3]. With the development nerve-sparing prostatectomy, the postoperative ED rate has reduced. In addition, the application of robot-assisted radical prostatectomy is beneficial in preserving erectile function [4,5]. However, these improved RP techniques have not been able to eliminate postoperative ED [6].

To clarify the mechanism of ED caused by neural injury and identify potential therapies, various animal models have been developed including the CN crush model, CN freeze model, CN dissection model, CN transection model and CN excision model [7–11]. These models represent different kinds of damage to the CN, all of which can reduce the number of neuronal nitric oxide synthase(nNOS)-containing nerve fibers and nNOS content in the corporal tissue [12]. In addition, CN injury might reduce the smooth muscle content and increase fibrosis by upregulating transforming growth factor β1 (TGF-β1)-Smad2/3 pathway in the corpus cavernosum [13]. CN injury can reportedly induce cell apoptosis by increasing the ratio of Bax to Bcl-2 or enhancing Caspase3 activity [14,15].

The CN crush model was widely used to mimic CN injury during nerve-sparing RP. However, many urologic surgeons now try to avoid crushing or even touching the CN in clinical practice. Even so, it is inevitable for surgeons to pull the CN directly during the procedures, or indirectly by pulling the adjacent tissue. We thereby attempted to develop a new CN injury model in rats, namely the CN traction model, and evaluate its effect on erectile function and the underlying mechanism, including nNOS expression, fibrosis, and apoptosis.

## Materials and methods

### Animal treatment

The study was approved by the Animal Care and Use Committee of Tongji Hospital, Tongji Medical College, Huazhong University of Science and Technology, Wuhan, China. All surgery was performed under anesthesia and all efforts were made to minimize suffering. Thirty-two male 8- week-old specific pathogen free Sprague–Dawley rats were kept under 12-hour light-and-dark cycles and fed with clean food and water by professional breeders. Rats were divided into four groups: control group, 1-minute CN traction group, 2-minute CN traction group, and 2-minute CN crush group.

**Fig 1:**
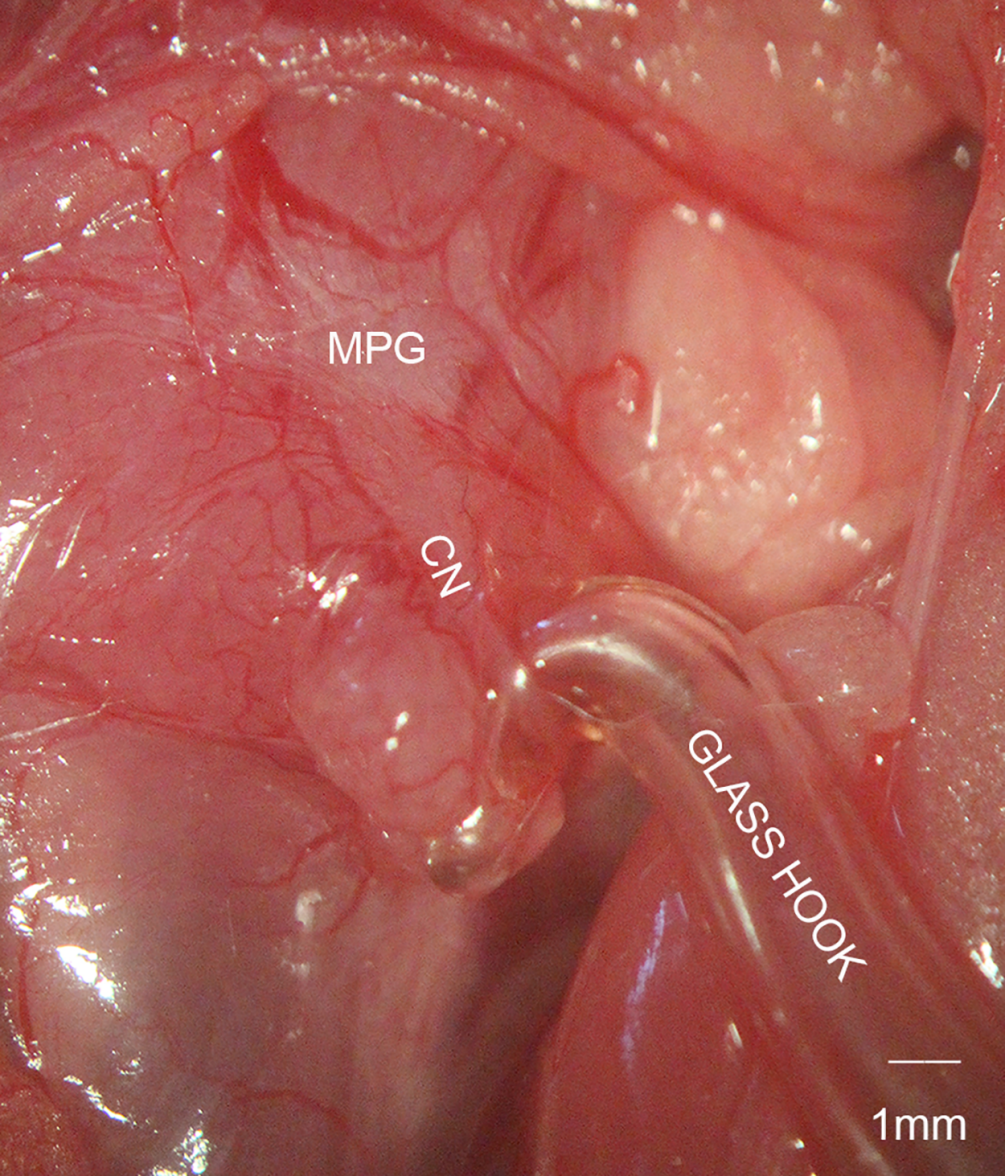
Cavernous nerve (CN) traction model. The CN is separated from the surface of prostate between the major pelvic ganglion (MPG) and apex of the prostate, and then pulled by a glass hook with a force of 0.2 Newton.

Before the surgery, rats were anesthetized with intraperitoneal injection of pentobarbital sodium (40mg/kg). The procedure started after confirming the anesthesia by tail pinch reflex. Fixed on the table, the rats were shaved and disinfected in the abdomen area. After performing a lower abdominal midline incision, we could see the bladder and prostate. Major pelvic ganglion (MPG) and CN were identified dorsolateral to the prostate with blunt dissection under a microscope. The MPG is a stellate ganglion, whereas the CN is derived from MPG and runs caudally in a groove between the rectum and urethra. Rats in the control group underwent only the MPG and CN exposure without further manipulation. In the CN traction group, bilateral CN between MPG and the apex of the prostate were dissected with micro-forceps and micro-scissors. The fascia covering the CN was cut by micro-scissors and the CN was separated from the prostate with micro-forceps. Then the CN was pulled using a glass hook (Fig 1), with a force of 0.2 Newton (N). The pulling force was determined by a high-precision tension dynamometer. The optimal force was determined by our preliminary experiment, which showed that 0.2N was the strongest force that would not break the CN. In the two CN traction groups, the pulling time was 1 min and 2 min separately. The CN crush was performed with a hemostatic clamp, which was applied to the CN about 5mm distal to the ganglion for 2 min with full tip closure. At last, the wounds were closed with sutures and were disinfected. After the procedure, the rats were placed in a warm and clean environment to recover from anesthesia. The first week after the surgery, rats were monitored everyday to see whether the wounds were infected or dehiscent. The food and water intake, as well as the mobilization of rats were also observed to evaluate the general health conditions.

### Evaluation of erectile function

Four weeks after the surgery, rats were anesthetized again with the same method. Intracavernosal pressure (ICP) was measured as described previously [16]. After anesthesia, a PE-50 tube containing heparinized saline (100 IU/mL) was inserted into the left carotid artery of the rats to monitor and record the mean arterial pressure (MAP). Then bilateral MPG and CN were exposed as described above. To measure the ICP, the penis was freed of skin and fascia, and the left penile crus was cannulated with a 25 G needle connected to a PE-50 tube attached to a pressure transducer. Electrical stimulation at different voltages (2.5V, 5 V, or 7.5V, 12HZ, 1.2ms, for 1 min) was applied with a bipolar hook electrode to the CN, distal to the ganglion but proximal to the CN traction point. A data acquisition system (PowerLab/4SP; AD Instruments, Bella Vista, Australia) was used to record the pressure data during the stimulation. The ratio between the maximal ICP and the corresponding MAP (ICP/MAP) was calculated and reported as a percentage.

After the procedure, the corpus cavernosum and the MPG were harvested. The midshaft of the penis was immediately fixed in 4% paraformaldehyde overnight and then embedded in paraffin for further histologic studies. The left cavernous tissue and the MPG were snap frozen in liquid nitrogen and then stored at −80°C for subsequent protein extraction.

### Western blot analysis

The cryopreserved corpus cavernosum or MPG was minced by scissors and homogenized by ultrasonic wave, followed by incubation in lysis buffer on ice for 30 min. 40μg of protein were loaded on 10% sodium dodecyl sulfate/polyacrylamide gels and transferred to polyvinylidene fluoride membranes (Millipore Corporation, Billerica, MA, USA). After being blocked with 5% bovine serum albumin (Sigma-Aldrich, St. Louis, MO, USA) for 2 h at room temperature, the membranes were incubated at 4°C overnight with antibodies against nNOS (1:500, BD Biosciences, San Jose, CA, USA), TGF-β1 (1:500, Abcam, Cambridge, UK), phospho-Smad2/3 (p-Smad2/3; 1:1,000, Cell Signaling Technology, Danvers, MA, USA), Smad2/3 (1:1000, Cell Signaling Technology, Danvers, MA, USA), Bcl-2 (1:1000, Affinity, Zhenjiang, China), Bax (1:1000, ProteinTech, Wuhan, China), or β-actin (1:500; Boster, Wuhan, China). Then the membranes were washed and incubated with horseradish peroxidase-labeled goat anti-mouse or goat anti-rabbit secondary antibody (1:10,000; Jackson ImmunoResearch, West Grove, PA, USA) at room temperature for 1 h. Protein bands were detected with the enhanced chemiluminescence detection system (Pierce; Thermo Fisher Scientific, Rockford, USA). Immunoblot bands were detected with the GeneGnome gel imaging system (Syngene, Cambridge, UK). The density value of each band was quantified with Image-Pro plus software (Media Cybernetics, Silver Spring, MD, USA).

### Histologic assessment

Immunofluorescence was used to determine the smooth muscle content in the cavernosum and nNOS content in the dorsal penile nerve. Penile tissue sections (5μm) were dewaxed in dimethylbenzene and then washed in phosphate buffered saline (PBS) three times. The slices were blocked by normal goat serum at room temperature for 1h and incubated with the antibody against α-smooth muscle actin (α-SMA; 1:100, Boster), nNOS (1:200, BD Biosciences) or neurofilament (1:200, ProteinTech) at 4°C overnight in a humidified and lightproof chamber. After three washes in PBS, the slices were incubated with DyLight-conjugated secondary antibodies (1:200, Abbkine, Redlands, CA, USA) for 1h at room temperature. Nuclei were stained with DAPI (Beyotime, Shanghai, China).

Masson’s trichrome staining was performed to assess the degree of cavernous fibrosis, which was determined by the ratio of collagen to smooth muscle in penile tissues of all groups.

Images were all obtained using fluorescence microscopy (Olympus, Tokyo, Japan) and analyzed by Image-Pro plus software (Media Cybernetics).

### Caspase3 activity analysis

Caspase3 activity in the penile tissue was detected by Caspase3 Activity Assay Kit (Beyotime) according to the manufacturer’s instruction. Briefly, tissues were grinded and then incubated in lysis buffer on the ice for 15 minutes. The mixture was subsequently centrifuged at 16 000g for 10 minutes at 4°C. The supernatant was collected and 2 mmol Ac-DEVD-pNA was added. The absorbance was measured at 405nm with a microplate reader (Thermo, MA, USA). Caspase3 activity of each sample was calculated according to the standard curve and normalized by the protein concentration.

### Statistical analysis

The results were analyzed using GraphPad Prism version 5.0 (GraphPad Software, San Diego, CA, USA) and were presented as mean ± standard deviation (SD). Statistical analysis was performed using one-way analysis of variance followed by the Tukey test for post hoc comparisons. Intergroup differences were considered significant at P < 0.05.

## Results

### Erectile function

**Fig 2:**
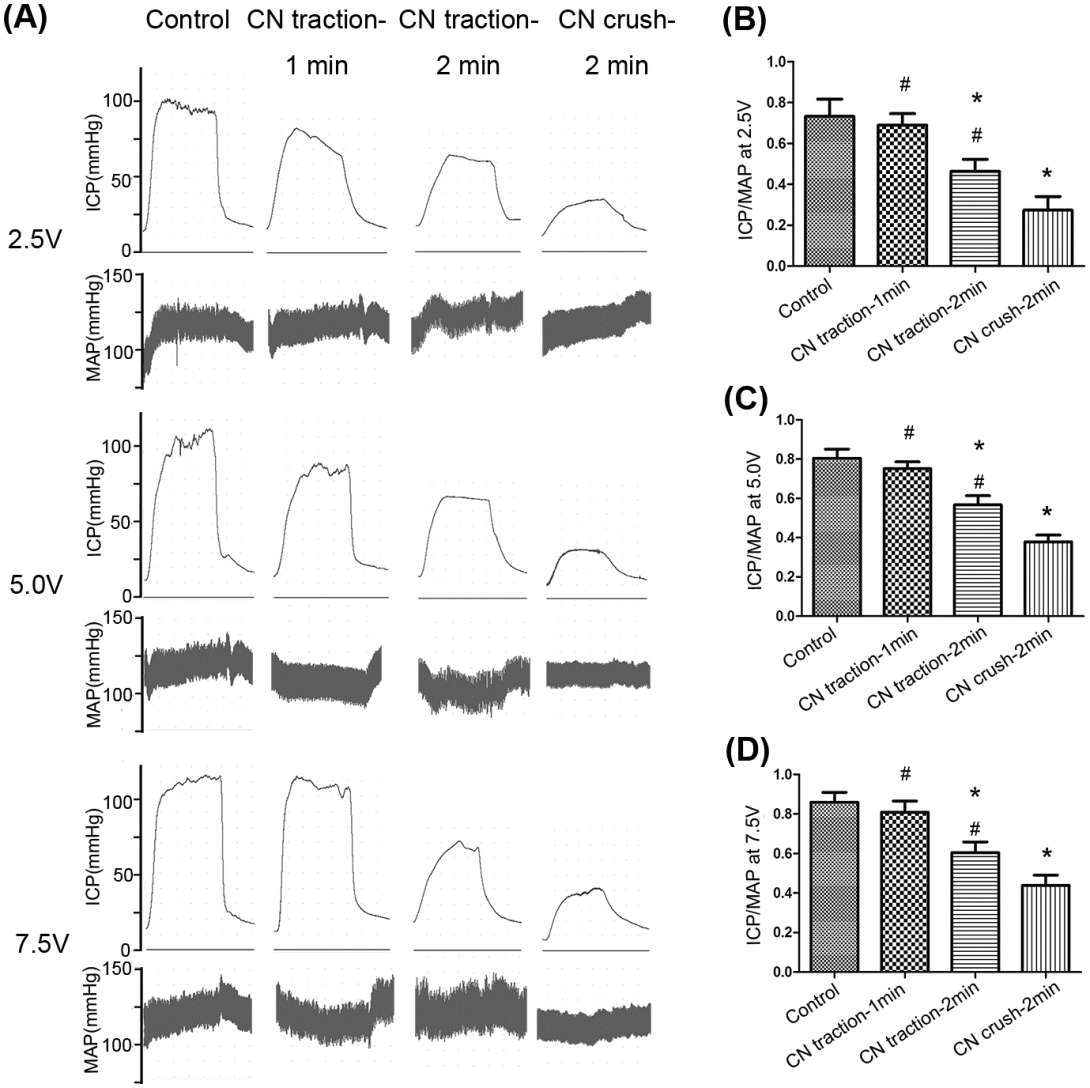
Comparison of erectile function in each group. (A) Representative recordings of intracavernosal pressure (ICP) and arterial pressure during 1-min electrical stimulation at 2.5V, 5.0V and 7.5V. (B,C,D) The ratio of ICP to mean arterial pressure (MAP) for each group, at 2.5V, 5.0V and 7.5V. N = 8 in each group. * P<0.05 compared with control group. # P<0.05 compared with 2-min CN crush group.

Erectile function was evaluated by ICP/MAP. Both kinds of CN injury caused damage to erectile function at different levels (Fig 2). The 1-min CN traction group showed no significant decrease in ICP/MAP compared with the control group at different levels of voltage (2.5, 5.0 and 7.5V, P>0.05 for each). However, the ICP/MAP in the 2-min CN traction group was lower than that of the control group and higher than that of the CN crush group (P<0.05, for each).

### Content of nerve fibers and nNOS

**Fig 3:**
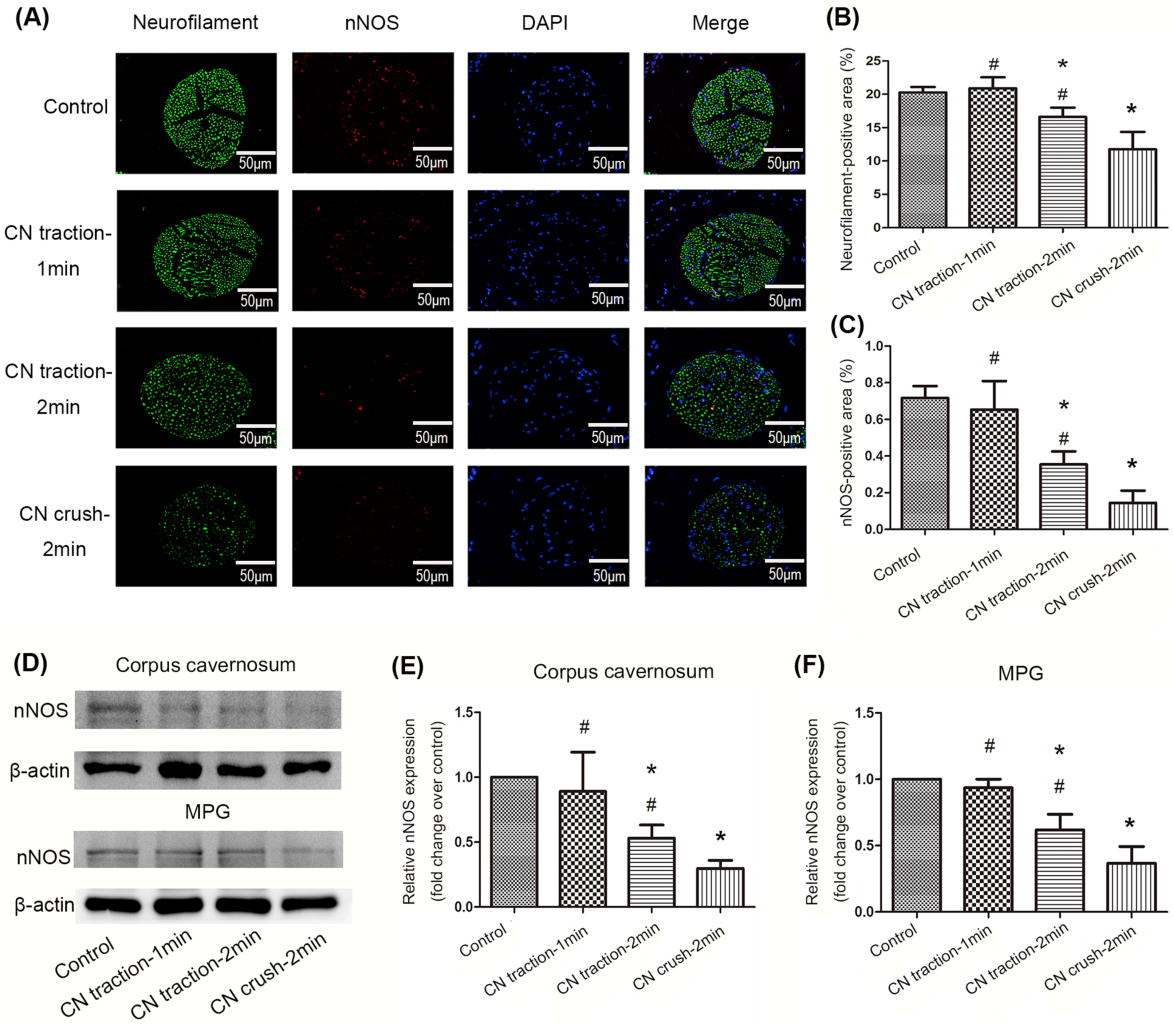
Changes of neuronal nitric oxide synthase(nNOS) and neurofilament content in the corpus cavernosum and MPG. (A) Neurofilament staining (green) and nNOS staining (red) in the dorsal penile nerve from different groups were performed with immunofluorescence. Nuclei were stained with DAPI (blue). Magnification: 400×. (B,C) Ratio of neurofilament positive area to the whole dorsal penile nerve area and nNOS positive area to the whole dorsal penile nerve area of four groups. Data are shown in the form of percentage. (D) Representative western blot bands for nNOS in the corpus cavernosum and MPG respectively. (E,F) Relative density of nNOS compared with β-actin in the corpus cavernosum and MPG. Data are shown as the fold changes over the control group. N = 6 in each group.* P<0.05 compared with control group. # P<0.05 compared with 2-min CN crush group.

Immunofluorescence staining showed that both the total number of nerve fibers (neurofilament positive)and the number of nNOS positive nerve fibers were reduced in the dorsal penile nerve in the 2-min CN traction group, compared with the control group (P<0.05). The 2-min CN crush group exhibited the lowest content of nerve fibers and nNOS among all groups (P<0.05, Fig 3A–3C). This was consistent with the results of western blot detecting nNOS expression in the corpus cavernosum (Fig 3D and 3E). In addition, 2-min CN traction also reduced the expression of nNOS in MPG (Fig 3D and 3F).

### Fibrosis in penile tissue

**Fig 4:**
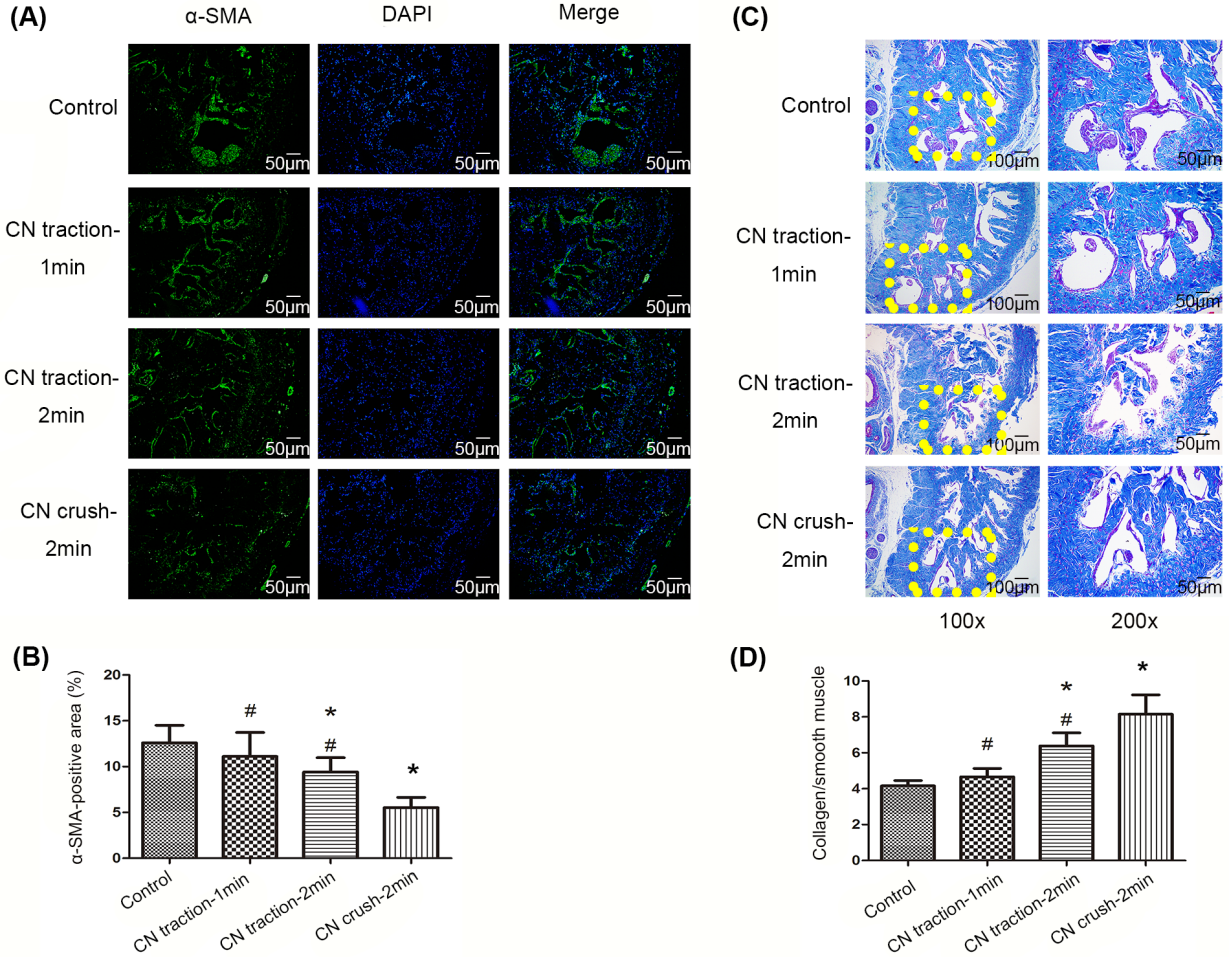
Changes of smooth muscle and collagen content in corpus cavernosum. (A) Smooth muscle staining (green) in corpus cavernosum in each group using immunofluorescence with anti-α-smooth muscle actin (α-SMA) antibody. Nuclei were stained with DAPI (blue). Magnification: 200×. (B) Ratio of α-SMA positive area to the whole cavernous tissue area. Data are shown in the form of percentage. (C) Masson’s trichrome staining of corpus cavernosum: smooth muscle cells are stained red, and collagen fibers are stained blue. (D) Ratio of collagen to smooth muscle, reflecting the level of fibrosis in penile tissue. N = 6 in each group. * P<0.05 compared with control group. # P<0.05 compared with 2-min CN crush group.

Immunofluorescence staining with anti-α-SMA antibody indicated that the content of smooth muscle in the corpus cavernosum was decreased in the 2-min CN traction group (P<0.05, Fig 4A and 4B). Moreover, the ratio of collagen to smooth muscle detected by Masson’s trichrome staining was higher in the 2-min CN traction group than in the control group (P<0.05, Fig 4C and 4D), which indicates that CN traction increased fibrosis in the penile tissue. However, the level of fibrosis was lower than that caused by CN crush (P<0.05).

**Fig 5:**
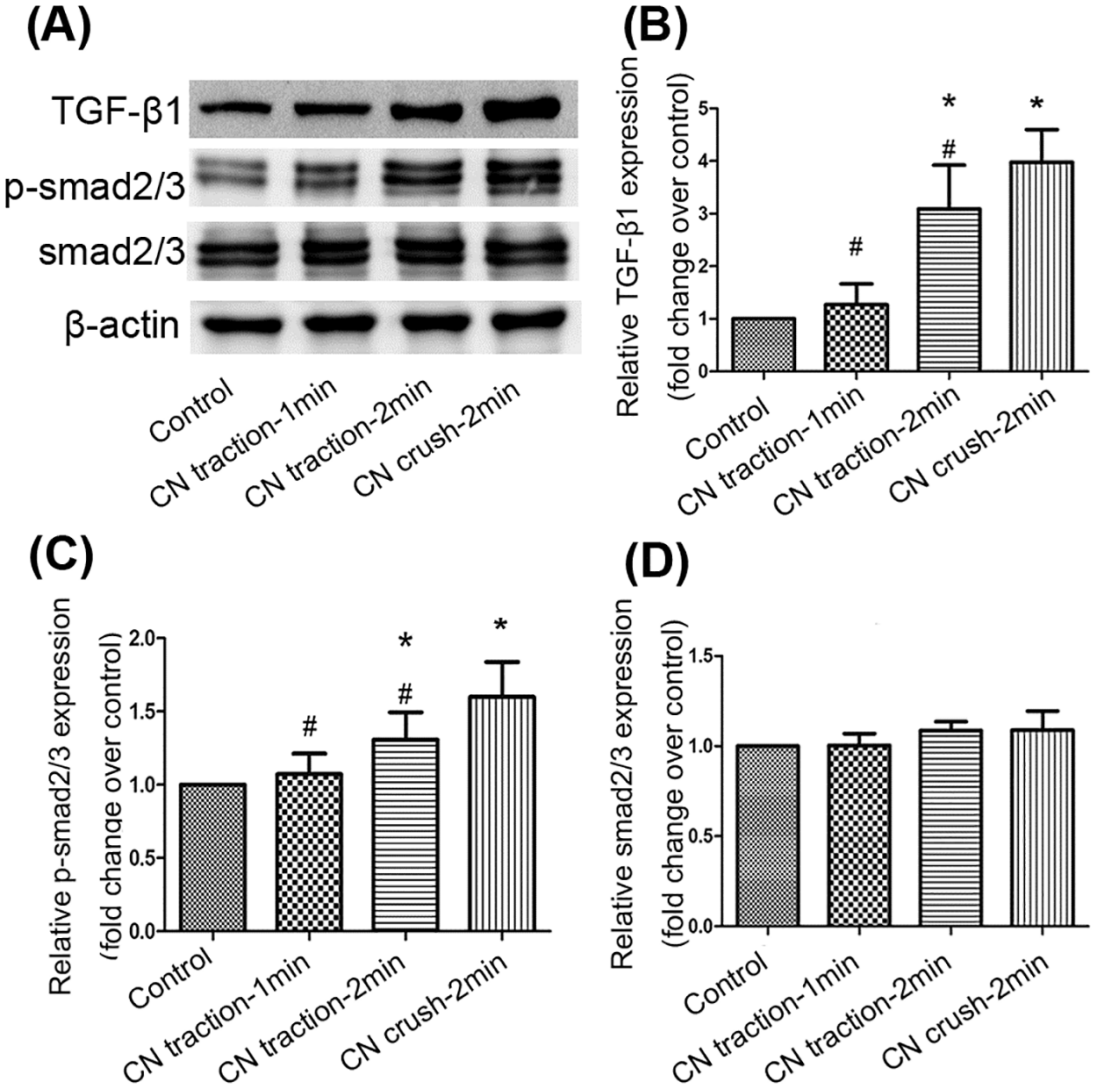
Changes of the transforming growth factor-β1 (TGF-β1)–Smad2/3 pathway in corpus cavernosum. (A) The expression of TGF-β1, Smad2/3 and phosphorylated Smad2/3 (p-Smad2/3) in penile tissue detected by western blot. (B,C,D) Relative density of TGF-β1, p-Smad2/3, and Smad2/3 compared with β-actin. Data are shown as the fold changes over the control group. N = 6 in each group. * P<0.05 compared with control group. # P<0.05 compared with 2-min CN crush group.

Western blot was used to detect the changes in TGF-β1–Smad2/3 signaling pathway in the cavernosum, whose activation could lead to fibrosis. TGF-β1 expression was markedly increased by CN traction (P<0.05, Fig 5A and 5B). The phosphorylation of Smad2/3 was also enhanced by CN traction (P<0.05, Fig 5C), whereas the expression of Smad2/3 showed no significant difference among the four groups (P>0.05, Fig 5D).

### Apoptosis in the corpus cavernosum

**Fig 6:**
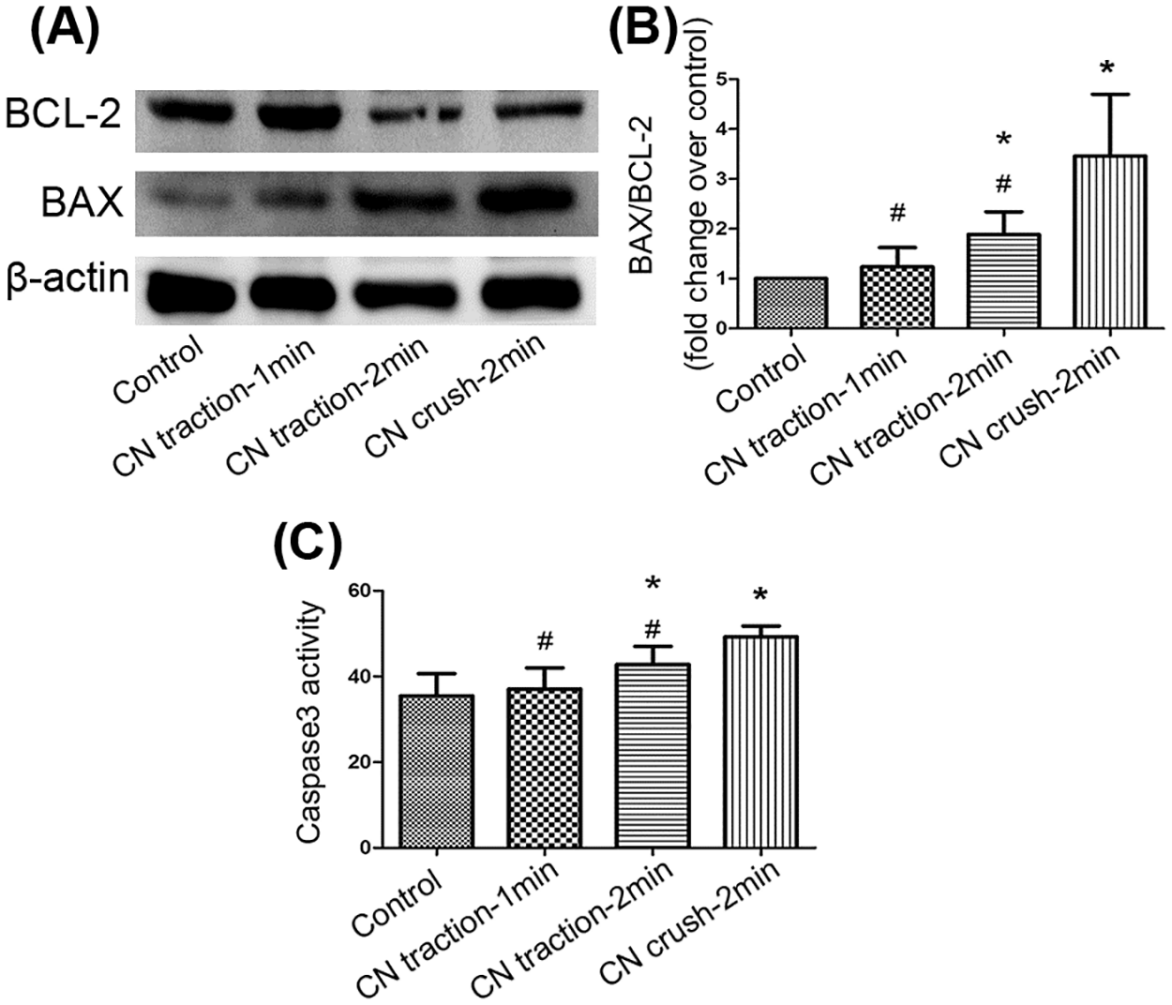
Changes of the apoptosis in corpus cavernosum. (A) The expression of Bcl-2 and Bax detected by western blot. (B) Ratio of Bax to Bcl-2. Data are shown as the fold changes over the control group. (C) Bar graph depicts caspase3 activity in penile tissues measured by a Caspase3 Activity Assay Kit, which was calculated according to a standard curve and normalized by the protein concentration. N = 6 in each group. * P<0.05 compared with control group. # P<0.05 compared with 2-min CN crush group.

Bcl-2 and Bax expression was measured by western blot and the ratio of Bax to Bcl-2 was calculated to indicate the level of apoptosis. Although 2-minute CN traction increased the ratio of Bax/Bcl-2, the ratio in the CN crush group was the highest (P<0.05, Fig 6A and 6B). Caspase3 activity was detected to further explore apoptosis in penile tissue. The results showed that 2-min CN traction enhanced caspase3 activity, compared with the control group (P<0.05, Fig 6C). Together, these data indicate that CN traction could lead to apoptosis in the corpus cavernosum.

## Discussion

In this study, we showed that traction to the CN within a short time does not cause apparent damage to erectile function, whereas traction lasting longer might lead to moderate ED. This indicates that CN traction is a relatively mild CN injury model. The underlying mechanism involved the reduction of nNOS expression in both MPG and the dorsal penile nerve, the decrease of smooth muscle content in the corpus cavernosum, and increased fibrosis and apoptosis.

ICP measurement is the most common technique for assessing erectile function in animal studies [1]. We performed the CN stimulation with three different voltages: 2.5V, 5V and 7.5V, which have all been used in previous studies [17–20]. It is possible that a maximum stimulation parameter might obscure the intergroup differences in erectile function [11]. However, the differences of erectile response among groups in our study were similar when using different voltages. The disparity between our study and the study from Jin HR et al. might result from the differences in the animals or the models used.

nNOS is a major NOS isoform that is responsible for the synthesis of nitric oxide and it plays a crucial role in the initiation of erection [21,22]. nNOS-positive nerve fibers innervating the penis derive mainly from the MPG [23]. Our results showed that the nNOS content in both the MPG and the dorsal penile nerve was reduced in the CN traction group, which is consistent with results in other CN injury models [9,24,25]. The number of neurofilament-positive nerve fibers in the dorsal penile nerve also declined, indicating that the axon contents were decreased after CN traction, which is also consistent with previous studies [26,27]. These findings suggest that CN traction could impair erectile function by reducing both the total number of nerve fibers and the number of nNOS-positive nerve fibers in the dorsal penile nerve.

Smooth muscle is an essential structural component in erectile tissue, which is responsible for the intracavernous hemodynamics. Its relaxation enables sufficient blood flow into the corpus cavernosum, thereby compressing the subtunical veins and preventing venous leakage. Ferrini MG et al.[28] have suggested that neuropraxias caused by CN injury might lead to a loss of corporal smooth muscle cells via apoptosis, which is the main cause of ED after CN injury. Another study has shown that injury to the CN can lead to the onset of apoptosis in the cavernosum and that most of the apoptotic cells are of smooth muscle origin [29]. Neural integrity plays a crucial role in maintaining the normal cavernous tissue structure because it can preserve the transmission of neurotrophic factors and maintain homeostasis within the cavernosum. In our study, we also found a decrease of smooth muscle content in the corpus cavernosum and increased apoptosis in the CN traction group.

In addition, Masson’s trichrome staining in this study showed that the ratio of collagen to smooth muscle content in corpus cavernosum was increased in the CN traction group, indicating increased fibrosis. Indeed, various studies have reported that CN injury can induce penile fibrosis [30–32]. On the one hand, fibrosis reduces the smooth muscle content in corpus cavernosum, and on the other hand, it disturbs the dilation of smooth muscle, thus leading to ED. TGF-β1 was believed to be a contributor to tissue fibrosis by promoting collagen synthesis [33]. TGF-β1 is a cytokine participating in various biological processes, including cell proliferation and differentiation, apoptosis, inflammation, and extracellular matrix formation [34]. After activation, TGF-β1 phosphorylates its downstream targets Smad2 and Smad3, which then regulate the transcription of fibrosis-related genes. Moreover, the TGF-β1–Smad pathway has also been reported to inhibit the proliferation of smooth muscle cells and endothelial cells [35,36]. In agreement with these findings, our work showed higher TGF-β1 expression and Smad2/3 phosphorylation in the CN traction group than in the control group.

To the best of our knowledge, the CN traction model has not been used or analyzed before. Here we found that CN traction is an effective way to impair erectile function in rats, and the injury it causes is not as severe as that caused by the CN crush model. The underlying mechanism might involve the reduced expression of nNOS, increased apoptosis of smooth muscle cells and fibrosis of the cavernosum.

A major limitation of our study is that we did not perform a time-dependent evaluation of the erectile function after the CN traction. Erectile function might vary over time after the surgery. Therefore, further study should be performed to investigate the erectile function changes at different time points after the CN traction procedure. Furthermore, the CN dissection model created by You D et al. [37], in which they dissected the CN with the micro-scissors and stretched the CN by turning the scissors at a right angle, resembles our model to some extent. However, compared with this CN dissection model, our model is more reproducible and easy to standardize among different laboratories because we used a tension dynamometer to determine and control the pulling force. Additionally, the study from You D and colleagues did not investigate the underlying mechanism, whereas we studied the detailed mechanism by which CN traction might cause ED.

## Conclusions

CN traction could lead to moderate ED by reducing the expression of nNOS in MPG and the dorsal penile nerve, increasing the apoptosis of smooth muscle cells, and enhancing penile fibrosis. CN traction is a relatively mild CN injury model, compared with the CN crush model. Other differences between CN traction and CN crush need further investigation.

## Supporting information

S1 Fig Supplemental file-raw blots. (DOCX)
