# Supplementary material for: The effect of cavernous nerve traction on erectile function in rats
Source: PLoS One. 2017 Oct 5;12(10):e0186077. doi: 10.1371/journal.pone.0186077 (PMC5628916; doi:10.1371/journal.pone.0186077)
Supplement: S1 Fig — (DOCX) [file pone.0186077.s001.docx]

**supplemental file-raw blots**


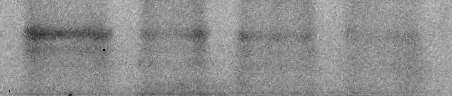
corpus cavernous-nnos


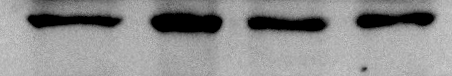
β-actin


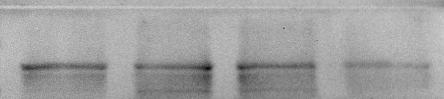
MPG-nnos


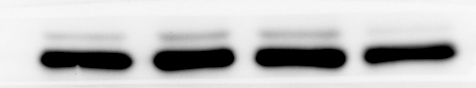
β-actin


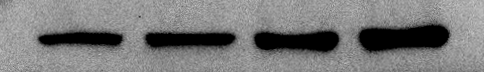
TGFβ-1


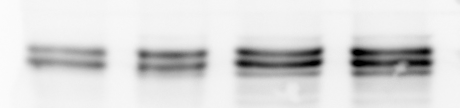
p-smad2/3


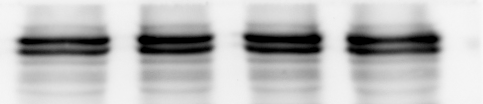
smad2/3


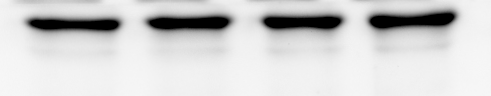
β-actin


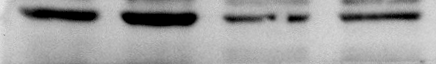
BCL-2


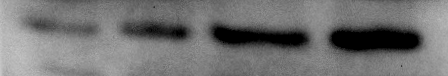
BAX


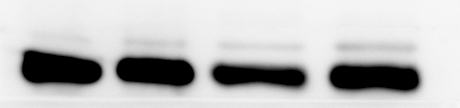
β-actin
